# Plasminogen Activator Inhibitor-1 in Skin Malignancies: Therapeutic Implications of Its Inhibition

**DOI:** 10.3390/biom15091317

**Published:** 2025-09-13

**Authors:** Taku Fujimura, Yusuke Muto, Yoshihide Asano

**Affiliations:** Department of Dermatology, Tohoku University Graduate School of Medicine, Sendai 980-8574, Japan; yusuk0610@derma.med.tohoku.ac.jp

**Keywords:** PAI-1, tumor microenvironment, SASP, melanoma, immune checkpoints, angiogenesis

## Abstract

Plasminogen activator inhibitor-1 (PAI-1), a key regulator of fibrinolysis, has emerged as a critical stromal factor that contributes to tumor progression in various malignancies, including skin cancers. Beyond its classical role in inhibiting plasminogen activators, PAI-1 exerts pleiotropic effects within the tumor microenvironment, promoting immunosuppression, angiogenesis, and extracellular matrix remodeling. This review highlights the tumor-promoting functions of PAI-1 in melanoma, cutaneous squamous cell carcinoma, cutaneous angiosarcoma and cutaneous T-cell lymphoma, with a particular focus on its modulation of tumor-associated macrophages, cancer-associated fibroblasts, and endothelial cells. We also discuss recent preclinical and clinical studies targeting PAI-1, including TM5614, a novel oral PAI-1 inhibitor currently under investigation in phase II /III trials. By summarizing the multifaceted roles of PAI-1 and its impact on the immune and stromal landscape of skin malignancies, this review provides a rationale for PAI-1 as a promising therapeutic target and calls for further clinical validation of PAI-1–directed therapies.

## 1. Introduction

Plasminogen activator inhibitor-1 (PAI-1) is a serine protease inhibitor that contributes to tumor-supportive mechanisms such as enhanced angiogenesis and tumor cell survival, thereby leading to unfavorable clinical outcomes in various malignancies, including cutaneous cancers [1,2,3]. Although PAI-1 has long been recognized for its role in thrombotic complications, such as deep vein thrombosis, through inhibition of urokinase-type and tissue-type plasminogen activators (uPA and tPA) [4], its biological functions extend far beyond the suppression of fibrinolysis.

PAI-1 is implicated in diverse pathological processes, including tissue fibrosis, atherosclerosis, and tumorigenesis [2]. Functionally, PAI-1 interacts strongly with vitronectin, thereby modulating cancer cell proliferation and motility [2]. Moreover, because PAI-1 has been reported to exert distinct effects on tumor angiogenesis and vascular formation depending on its concentration, it has been termed a “Janus-faced” molecule [5]. At low concentrations, PAI-1 exerts cytoprotective effects by shielding normal endothelial cells (ECs) from Fas ligand-mediated apoptotic injury under stress conditions such as detachment, vascular endothelial growth factor (VEGF) exposure, or hypoxia, thereby promoting angiogenesis [5]. In contrast, at high concentrations, PAI-1 strongly inhibits uPA/tPA activity, leading to reduced plasmin generation. Consequently, extracellular matrix (ECM) degradation becomes insufficient, impairing endothelial cell invasion and tube formation, thereby suppressing angiogenesis [5,6]. This results in a hypoxic tumor microenvironment, which in turn suppresses antitumor immunity and contributes to tumor progression [1,2,5,6]. Collectively, these findings support the so-called “PAI-1 paradox,” wherein serum PAI-1 levels correlate with tumor prognosis [1,2,7]. Such concentration-specific effects highlight the complex and context-dependent role of PAI-1 within the tumor microenvironment.

In addition to its function as an angiogenic regulator, PAI-1 also promotes recruitment of tumor-associated macrophages (TAMs) within the tumor microenvironment (TME) by upregulating focal adhesion kinase (FAK) expression [7]. Furthermore, PAI-1 influences immune evasion by regulating programmed death-ligand 1 (PD-L1) expression through the JAK/STAT signaling pathway in both tumor cells and stromal components, including TAMs and cancer-associated fibroblasts (CAFs) [3,8,9]. In this review, we comprehensively examine the multifaceted roles of PAI-1 across different types of cutaneous malignancies.

## 2. PAI-1 in Skin Cancers

Accumulating evidence suggests that PAI-1 is a promising molecular target for treating various malignancies, including melanoma. To date, two clinical studies have been completed [10,11], and two additional trials are ongoing [12,13].

### 2.1. Malignant Melanoma

Unlike in Caucasian populations, the therapeutic efficacy of anti-PD-1 antibodies (Ab) in East Asian patients has been shown to be lower in several real-world clinical studies [14,15]. In Japan, for example, the objective response rate (ORR) to anti-PD-1 Ab therapy for acral melanoma was 16.6%, and the median overall survival (OS) was 18.1 months, both of which were significantly lower compared with Caucasians [15]. Furthermore, Bai et al. retrospectively collected and analyzed clinical data from 1135 patients with unresectable or advanced melanoma who received anti-PD-1 Ab monotherapy between 2009 and 2019 across five independent institutions in the United States, Australia, and China [15]. The analysis revealed an ORR of 54% (95% CI: 50–57) in Caucasians, whereas East Asians, Hispanics, and African Americans demonstrated an ORR of 20% (95% CI: 13–28) [15]. The median progression-free survival (PFS) was 14.2 months (95% CI: 10.7–20.3) in Caucasians, but only 5.4 months (95% CI: 4.5–7.0) in East Asian, Hispanic, and African American patients, indicating lower efficacy of anti-PD-1 antibodies in these groups for non-acral melanoma as well (adjusted *p* < 0.001) [15]. Of note, in acral melanoma, no significant differences in ORR and PFS were observed between Caucasians and East Asian, Hispanic, or African American patients [15]. Single-cell RNA sequencing further revealed that, compared with cutaneous melanoma, acral melanoma harbors fewer tumor-reactive CD8 clusters and a higher proportion of regulatory T cells (Tregs) with direct tumor-recognition capacity, suggesting the establishment of an immunosuppressive TME in acral melanoma [16].

Taken together, the efficacy of immune checkpoint inhibitors (ICIs) for unresectable melanoma in East Asia tends to be lower than in Caucasians [14,15]. Potential explanations include the higher prevalence of acral melanoma [17], the low tumor mutational burden (TMB) associated with acral melanoma [18], and profound T-cell exhaustion within the acral melanoma TME [16]. Thus, both tumor-intrinsic factors (e.g., low TMB) and host immune dysfunction (e.g., T-cell exhaustion) likely contribute to reduced ICI efficacy in East Asian melanoma patients. In considering combination strategies to improve ICI efficacy in non-Caucasian melanoma, restoration of host T-cell function is particularly critical. Ipilimumab enhances anti-PD-1 Ab efficacy by expanding tumor-infiltrating tumor-reactive T cells [19]. However, when alternative immunosuppressive pathways are present, the clinical benefit may remain limited. Therefore, strategies such as further checkpoint blockade with agents like relatlimab [20], inhibition of tumor-promoting signals in the TME (e.g., IL4I1 and AhR signaling) [21], or molecular targeted therapies such as BRAF and MEK inhibitors [22], are expected to improve outcomes.

**Figure 1:**
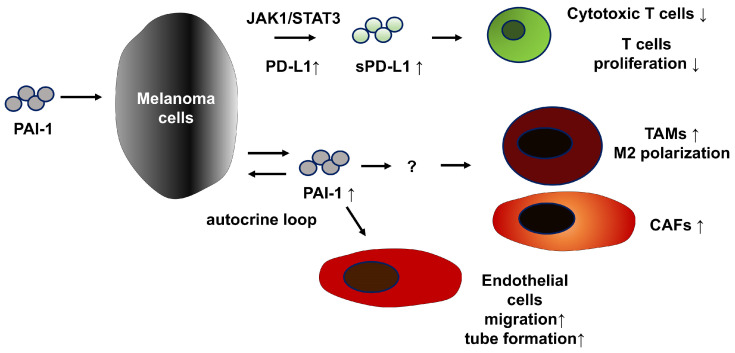
The multiple tumor-promoting functions of PAI-1 in melanoma.

Plasminogen activator inhibitor-1 (PAI-1) is frequently expressed in both primary and metastatic melanoma lesions, with particularly high expression in cutaneous metastases correlating with increased invasiveness and motility, implicating PAI-1 in risk stratification for metastasis [23]. Moreover, PAI-1 expression in melanoma cells and elevated serum PAI-1 levels in patients positively correlate with the efficacy of anti-PD-1 antibody therapy [3]. Intriguingly, in B16F10 melanoma cells, PAI-1 promotes the release of soluble PD-L1 through JAK1/TYK2–STAT3 signaling, thereby suppressing CD8^+^ cytotoxic T-cell activity in a JAK-dependent manner [9]. PAI-1 inhibition reduces immunosuppressive cells within the TME, such as M2-type TAMs and CAFs, thereby augmenting the antitumor efficacy of anti-PD-1 Ab [9]. In addition, in the human melanoma model A375, PAI-1 inhibition suppresses endothelial cell migration and tube formation, thereby limiting tumor angiogenesis and exerting antitumor effects [24]. Collectively, these findings suggest that PAI-1 contributes to melanoma progression through multiple mechanisms, and its inhibition may represent a promising therapeutic strategy for advanced melanoma (Figure 1).

In fact, the PAI-1 inhibitor TM5614 has demonstrated promising results in patients with unresectable melanoma resistant to anti-PD-1 Ab such as nivolumab (Trial ID: jRCT2021210029) [10]. In a per-protocol analysis of 27 patients, the ORR at 8 weeks with TM5614 plus nivolumab combination therapy was 25.9% (95% CI: 12.9–44.9%; *p* = 0.027). This efficacy is comparable to the reported ORR of 28–31% for nivolumab plus ipilimumab combination therapy in Western populations [25,26], and substantially higher than the ORR of 16.1% observed in Japanese patients treated with the same regimen in later-line settings [27]. Despite the relatively short treatment duration of only 8 weeks, the median PFS reached 174 days (95% CI: 114.4–232.9), suggesting that TM5614 may serve as a viable strategy to overcome PD-1 inhibitor resistance. Moreover, post hoc analyses of the TM5614-MM trial revealed marked reductions in serum IL-4 levels in responders, as well as decreased circulating L-tryptophan concentrations compared with non-responders [28]. Based on these findings, an investigator-initiated phase III randomized, placebo-controlled, double-blind trial (jRCT2021240049) evaluating the efficacy and safety of TM5614 plus nivolumab in patients with unresectable melanoma refractory to anti-PD-1 monotherapy is currently underway.

PAI-1 suppresses the proliferation and function of cytotoxic T cells as well as promotes the induction of TAMs and CAFs in the TME. PAI-1 also promotes the migration and tube formation of ECs in melanoma.

### 2.2. Cutaneous Squamous Cell Carcinoma (cSCC)

**Figure 2:**
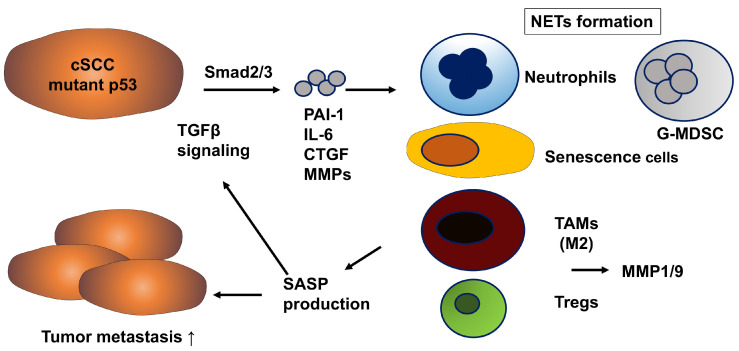
PAI-1 promotes metastasis in cSCC.

In cSCC, PAI-1 expression is markedly elevated compared to basal cell carcinoma and actinic keratosis, and has long been implicated in tumor invasion and metastasis [29]. Upregulation of PAI-1 is considered an early event in SCC progression and is localized in tumor cells and myofibroblasts at the invasive front of cSCC lesions [30]. In cSCC, mutant p53 (mutp53) promotes tumor progression by enhancing Smad2/3 transcriptional activity, thereby inducing the expression of TGF-β1-responsive genes such as PAI-1, connective tissue growth factor (CTGF), and IL-6 [31]. Indeed, mutp53 expression correlates with PAI-1 overexpression in clinical cSCC samples [31]. Furthermore, analysis of public databases reveals that mutp53, activation of the TGF-β signaling pathway, and high PAI-1 expression are associated with higher tumor malignancy and poor prognosis [31]. Notably, many TGF-β1-responsive genes including PAI-1, IL-6, CTGF, and matrix metalloproteinases (MMPs) are components of the senescence-associated secretory phenotype (SASP), which reprograms the behavior of stromal cells in the TME [32,33]. For example, PAI-1 activates the p53/p21 pathway, promotes cellular senescence via SASP induction, and senescent cells secrete SASP factors such as IL-6 and MMPs that recruit M2-type TAMs and regulatory T cells (Tregs), thereby establishing an immunosuppressive TME and ultimately accelerating tumor progression [34,35] (Figure 2). Interestingly, cSCC arising from hidradenitis suppurativa (HS), a neutrophilic autoinflammatory skin disease, has a significantly worse prognosis than cSCC induced by sun exposure [36]. This may be due to the involvement of multiple SASP factors such as IL-17 and IL-1 families [36]. Among these, IL-8—known as a SASP component—has recently attracted attention not only for its conventional role in neutrophil recruitment, but also for its central role in cancer malignancy by promoting metastasis and treatment resistance through the formation of an inflammatory TME [37]. Notably, tumor-derived IL-8 has been shown to induce the formation of neutrophil extracellular traps by granulocytic myeloid-derived suppressor cells, thereby contributing to immunosuppression and metastasis [38].

It has been demonstrated in many models that SASP induces PAI-1, and furthermore, PAI-1 itself enhances SASP production, thereby establishing a positive feedforward loop between SASP and PAI-1 (Figure 2). PAI-1 suppresses the abscopal effect of radioimmunotherapy by inducing the differentiation of pericytes into SERP2 high-expressing CAFs via the LRP1/p65 signaling pathway in multiple human and murine cancer types [39]. In addition, PAI-1-producing TAMs promote oxaliplatin resistance in colorectal cancer, while cisplatin activates CAFs in esophageal squamous cell carcinoma to increase PAI-1 secretion, thereby conferring cisplatin resistance [40]. Taken together, these findings suggest that inhibition of PAI-1 in cSCC may enhance the abscopal effect of radioimmunotherapy and prevent resistance to cytotoxic chemotherapeutic agents, indicating that PAI-1 could represent a promising therapeutic target in cSCC.

In cSCC, tumor-derived PAI-1 recruit immunosuppressive cells such as granulocytic myeloid-derived suppressor cells, neutrophils, TAMs, and Tregs, leading to promoting metastasis of cSCC.

### 2.3. Cutaneous Angiosarcoma (CAS)

CAS is characterized by malignant transformation of endothelial cells (ECs), frequently exhibiting high expression of angiogenesis-related growth factors and receptors, such as VEGFR1/2/3 [41,42]. Accordingly, multitargeted tyrosine kinase inhibitors (TKIs) such as pazopanib, which block these angiogenic signaling pathways, have been employed in the treatment of soft tissue sarcomas including angiosarcoma, although their efficacy remains limited [43,44]. In fact, in the phase II OER-073 trial evaluating the safety and efficacy of pazopanib in angiosarcoma, the ORR was only 3%, with a clinical benefit rate of 48%, median PFS of 14.4 weeks, and a 3-month PFS rate of 54.6% (95% CI: 36.0–82.9) [43]. To enhance the therapeutic efficacy of pazopanib, a phase II TAPPAS trial investigated the combination of pazopanib with the anti-endoglin antibody carotuximab (TRC105), which targets Endoglin (CD105), highly expressed in tumor endothelium. However, no improvement in PFS, OS, or ORR was observed in advanced angiosarcoma [44].

PAI-1 has also been identified as a critical factor in the pathogenesis of cutaneous angiosarcoma (CAS), with high expression levels correlating with poor prognosis [45]. Mechanistically, PAI-1 promotes tumor angiogenesis by enhancing the production of pro-angiogenic mediators such as IL-23p19, VEGF-C, CXCL5, and CCL20 [45]. Moreover, PAI-1 and PAI-1-induced VEGF signaling enhance EC survival by inhibiting Fas ligand-mediated apoptosis [5]. Importantly, PAI-1 also activates the p53/p21 pathway in ECs, thereby inducing SASP (senescence-associated secretory phenotype) and promoting cellular senescence [32]. Indeed, PAI-1 overexpression in human umbilical vein endothelial cells (HUVECs) induces increased SA-β-gal activity, activation of p53, p21, p16, and Rb, and ultimately cellular senescence [34]. Furthermore, SASP factors (IL-6, IL-8, MMP3) secreted from these senescent HUVECs act on surrounding cells such as tumor-associated macrophages (TAMs) and cancer-associated fibroblasts (CAFs), thereby facilitating tumorigenesis [35]. These findings have recently highlighted the importance of the tumor immune microenvironment in soft tissue sarcomas such as CAS. Propranolol has been shown to exert therapeutic effects by modulating TAMs and tumor-infiltrating lymphocytes (TILs) [46]. Since CAS expresses β1/β2/β3-adrenergic receptors, high-dose propranolol induces tumor cell death, thereby exerting antitumor effects [47]. Moreover, β2-adrenergic receptor signaling suppresses CD8^+^ T-cell responses while increasing IL-6 and IL-8 production; thus, propranolol is thought to inhibit this inflammatory pathway, preventing TAM recruitment and contributing to antitumor efficacy [47]. As IL-6 represents a canonical SASP factor, suppression of TAMs through modulation of SASP signaling may further underlie the antitumor effects in CAS. In addition, PAI-1 expression induced by SASP has been reported in multiple models [32], suggesting that PAI-1 may amplify SASP production and promote tumor progression through a SASP–PAI-1 feed-forward loop. This provides a rationale for why VEGFR1/2/3 inhibition by multitargeted TKIs alone is insufficient for therapeutic efficacy. Based on these insights, targeting PAI-1 may represent a promising therapeutic strategy to control tumor growth in CAS. Currently, a phase II clinical trial is ongoing to evaluate the efficacy and safety of TM5614 in combination with paclitaxel in this rare but highly aggressive vascular malignancy (jRCT2021230016) [12].

### 2.4. Mycosis Fungoides (MF), Cutaneous T Cell Lymphoma (CTCL)

Mycosis fungoides (MF) is an indolent subtype of cutaneous T-cell lymphoma (CTCL) [48], and its early lesions resemble atopic dermatitis (AD) [49]. In recent years, in addition to its long-established role in stratum corneum barrier function, hyaluronic acid (HA) has begun to attract attention as an immunomodulatory factor in AD [50]. Cytokines and chemokines induced by HA include numerous SASP-related mediators such as IL-1, IL-6, IL-8, IL-17, TNF-α, and CXCL9/10, many of which overlap with those found in early MF lesions [51]. Consequently, the role of HA in MF has also garnered increasing interest [52,53]. Indeed, in tumor-stage MF, HA predominantly exists in its low-molecular-weight form (LMWHA), contributing to the establishment of an immunosuppressive tumor microenvironment (TME) through interactions with tumor-associated macrophages (TAMs) and cancer-associated fibroblasts (CAFs) [53]. Previous studies have shown that bexarotene suppresses tumor growth by inhibiting HA synthesis [53]. This effect is mediated by reduced binding of retinoid X receptor α (RXRα) to the promoter regions of hyaluronic acid synthases (HAS1 and HAS2) in both tumor cells and fibroblasts [53,54]. Taken together, these findings suggest that bexarotene, which has been widely used as a therapeutic agent for MF from UV-resistant lesions to tumor stage [48], may exert its therapeutic efficacy, at least in part, through suppression of SASP.

As noted above, SASP induces PAI-1 expression, while PAI-1 in turn amplifies SASP production, forming a SASP–PAI-1 feed-forward loop that promotes tumor progression. This mechanism has been implicated in other malignancies as well as skin cancer [32], and its involvement has also been suggested across various stages of MF. Indeed, in tumor-stage MF, serum PAI-1 levels and downstream SASP factors such as MMP-9 are elevated compared with early lesions [55]. Interestingly, these SASP factors are reduced in patients responding to bexarotene treatment, suggesting their contribution to disease activity [55]. Mechanistically, bexarotene increases the expression of PPARγ and SIRT6 while downregulating phosphorylated FoxO3a and broadly suppressing SASP factors (IL-1β, IL-6, and TNF-α), thereby attenuating neutrophil-driven inflammation [56]. In addition, in CTCL models, bexarotene has been shown to downregulate the SASP factor IL-23p19 [55], indicating that bexarotene may mitigate SASP-related inflammation in MF. Since SASP has been reported to activate PAI-1 signaling and contribute to various senescence-associated pathologies [32], HA accumulation in early MF may promote PAI-1 expression through SASP induction. This SASP–PAI-1 interaction may drive progression to the tumor stage [55]. Therefore, targeting PAI-1 could suppress SASP production, enhance antitumor immunity, and represent a promising therapeutic strategy for MF.

The tumor-promoting effects of PAI-1 in different cancer types, as well as the therapeutic agents expected to exhibit synergistic efficacy when combined with PAI-1 inhibitors, are described below (Table 1).

## 3. Diverse Tumor-Promoting Effects of PAI-1 in the TME

Over the past few decades, immunotherapy has become a leading treatment option for advanced skin cancers [44]. Antibodies targeting PD-1 have been established as a central component in the management of advanced melanoma [45]. When used in combination with ipilimumab, these agents markedly improve survival outcomes in patients with unresectable melanoma [45]. Nonetheless, the therapeutic benefit of immunotherapy differs across ancestral groups and appears particularly limited in Asian patients with melanoma [14,15,57,58,59,60]. This reduced efficacy has been linked in part to the predominance of acral melanoma with a low tumor mutation burden (TMB) in Asian populations, though recent evidence suggests that additional mechanisms are involved [14,15]. For example, Bai et al. demonstrated that distinct TME subtypes are strongly associated with resistance to immune checkpoint inhibitors (ICIs) [60]. These findings underscore the critical role of TILs and the stromal components that regulate their activity within the TME. Given the central importance of TILs in mediating responses to ICIs, a comprehensive evaluation of stromal elements—such as TAMs, CAFs, and extracellular matrix (ECM) proteins—is warranted to better predict immunotherapy outcomes in skin cancers [60]. Factors including PAI-1, IL-4, and the TGF-β–induced ECM protein periostin (POSTN) have been shown to influence TAM polarization [61]. These reprogrammed TAMs subsequently secrete a range of chemokines and angiogenic factors, which vary by tumor site and contribute to the maintenance of an immunosuppressive TME in multiple cancer types [61].

### 3.1. Immunomodulatory Role of PAI-1 in TAM Regulation Within the TME

TAMs are abundantly present in skin cancers and, together with stromal components such as CAFs and Tregs, contribute to tumor progression by maintaining an immunosuppressive TME [61,62,63,64]. Although TAMs represent a heterogeneous population, they are often M2-polarized macrophages differentiated under the influence of tumor stromal immune cues that activate the JAK/ STAT3 signaling pathway [61,62,63,64,65,66,67]. For instance, in murine myeloid cells, deficiency of the m6A methyltransferase METTL3 activates STAT3 and NF-κB signaling, promotes the expression of M2-associated genes, and enhances tumor growth and metastasis in B16F10 melanoma [65]. Moreover, METTL3 deficiency attenuates the efficacy of anti-PD-1 Ab therapy, indicating that STAT3 activation in myeloid cells mediates resistance to ICIs [65]. Similarly, inhibition of HDAC6 suppresses M2 polarization through STAT3 inactivation, thereby reducing tumor burden in SM1 melanoma and humanized NSG-SGM3 mouse models [66]. As noted earlier, dermal fibroblast-derived low-molecular-weight HA promotes TAMs differentiation into the M2 phenotype in MF [53]. Intriguingly, PAI-1 also drives TAMs M2 polarization through the JAK/STAT3 pathway, thereby sustaining an immunosuppressive environment in melanoma [9]. These findings collectively underscore that STAT3-mediated M2 TAM polarization plays a central role in maintaining the immunosuppressive TME through multiple mechanisms.

As a stromal-derived factor regulating TAM function, PAI-1 has attracted attention as a potential therapeutic target in various malignancies [3,8,62,64,68,69]. In melanoma, PAI-1 promotes PD-L1 expression in both tumor cells and stromal cells via the JAK1/STAT3 pathway [9]. Pharmacological inhibition of PAI-1 reduces immunosuppressive cell populations such as TAMs and CAFs, enhances infiltration of cytotoxic T cells, and, in combination with anti-PD-1 Ab therapy, induces tumor regression [9]. Furthermore, extracellular PAI-1 can induce clathrin-dependent internalization of PD-L1, thereby reducing its surface expression [8]. In addition, PAI-1 enhances the secretion of SASP factors such as CXCL5 from M2 macrophages [3]. Beyond melanoma, PAI-1 facilitates tumor progression in other cancers by promoting M2 polarization through JAK2/STAT3 signaling [65]. In gastric cancer, single-cell RNA sequencing has revealed that high intratumoral PAI-1 expression correlates with advanced stage, poor prognosis, and increased M2 macrophage infiltration [62]. In colorectal cancer, PAI-1, regulated by FGF/FGFR2 signaling, induces M2 polarization and is associated with unfavorable clinical outcomes [63]. Mechanistically, PAI-1 recruits monocytes through its N-terminal LRP1-binding domain and promotes M2 differentiation via IL-6/STAT3 autocrine signaling mediated by its C-terminal uPA-binding domain [69]. High PAI-1 expression is also associated with TAM accumulation and poor prognosis in head and neck squamous cell carcinoma [68].

Taken together, these findings demonstrate that PAI-1 is a key driver of TAM recruitment and M2 polarization in the TME through JAK/STAT3-dependent mechanisms. By inducing monocyte infiltration via SASP factors such as IL-6 and CXCL5, PAI-1 sustains an immunosuppressive TME, thereby facilitating tumor progression across a range of malignancies, including skin cancers.

### 3.2. Significant Effects of PAI-1 on Cancer-Associated Fibroblasts (CAFs)

CAFs are a heterogeneous population of stromal cells that produce tumor-promoting factors across many cancer types, including cutaneous malignancies [70,71]. They characteristically express high levels of α-smooth muscle actin (α-SMA), together with activation markers such as fibroblast activation protein (FAP), platelet-derived growth factor receptor (PDGFR), and periostin, thereby exerting functions such as tumor promotion and angiogenesis [70]. Given their heterogeneity, the expression of CAF-related markers varies depending on the cancer type, and their tumor-promoting mechanisms may differ across skin cancers [70,71]. For instance, in advanced melanoma, α-SMA^+^ CD90^+^ FAP^+^ fibroblasts have been implicated in the therapeutic efficacy of anti-PD-1 Ab monotherapy [72]. Furthermore, these α-SMA^+^ CD90^+^ FAP^+^ fibroblasts significantly correlate with survival outcomes in melanoma patients treated with anti-PD-1 antibodies [72].

Forsthuber et al. further subclassified CAFs in skin cancers into three groups: myofibroblast-like RGS5^+^ CAFs, matrix CAFs (mCAFs), and immunomodulatory CAFs (iCAFs) [73]. Their report demonstrated that mCAFs dominate in low-grade cutaneous malignancies (e.g., basal cell carcinoma), whereas both mCAFs and iCAFs prevail in highly malignant cancers such as melanoma and invasive cSCC. Tumors dominated by mCAFs exhibit reduced T-cell infiltration, which may compromise immunotherapy efficacy. Conversely, iCAF-enriched tumors produce CXCL1 and CXCL8, which recruit neutrophils, monocytes, and other SASP-associated immune cells, thereby fostering a immunosuppressive TME. These findings suggest that CAFs may represent promising targets for immunomodulatory therapies [74,75].

Although the precise mechanisms through which PAI-1 drives tumor progression via CAFs remain incompletely understood, multiple studies have underscored the critical role of CAF-derived or CAF-activated PAI-1 [40,76,77,78]. As noted above, in several cancers, including melanoma, lung cancer, and colorectal carcinoma, radiotherapy induces the release of PAI-1 into the circulation, which acts at distant sites to drive the differentiation of pericytes into SFRP2-high CAFs through the LRP1/p65 signaling pathway. This process establishes an immunosuppressive perivascular niche that hinders intratumoral infiltration of CD8^+^ T cells [78]. Importantly, analysis of patient cohorts in this study revealed that elevated plasma PAI-1 levels are associated with reduced efficacy of radioimmunotherapy [78]. These results indicate that PAI-1-induced SFRP2 ^high^ CAFs play a central role in suppressing the abscopal effect of radioimmunotherapy, suggesting that therapeutic strategies targeting SFRP2 or PAI-1 may enhance abscopal responses and improve clinical application of radioimmunotherapy. In lung cancer, PAI-1 has been shown to promote chemotherapy resistance (e.g., to cisplatin) and tumor progression via CAFs [77]. In cervical SCC, CAF-derived PAI-1 induces endothelial-to-mesenchymal transition (EndoMT) in lymphatic endothelial cells, thereby enhancing lymphangiogenesis and facilitating lymphatic metastasis [76]. In hepatocellular carcinoma, tumor cells induce CAFs that drive M2 polarization of TAMs, which in turn secrete PAI-1, forming an autocrine loop that promotes an immunosuppressive TME [78]. Collectively, these findings suggest that PAI-1 facilitates lymphangiogenesis, chemotherapy resistance, and ICI resistance through CAFs, ultimately contributing to poor patient outcomes.

## 4. Future Perspective

The growing body of evidence implicating PAI-1 as a central orchestrator of tumor–stroma interactions in skin malignancies underscores its potential as a therapeutic target. By modulating the activity of TAMs, CAFs, and other components of the TME, PAI-1 promotes immune evasion, angiogenesis, lymphangiogenesis, and resistance to both cytotoxic chemotherapy and ICIs. These multifaceted roles position PAI-1 inhibition as a strategy that could complement existing treatments across a broad spectrum of skin cancers, including melanoma, cSCC, CAS, and CTCL.

Despite encouraging preclinical data and promising early-phase clinical trial results—particularly with the small-molecule inhibitor TM5614—several challenges remain. First, the dual and context-dependent effects of PAI-1 on tumor progression and angiogenesis suggest that optimal dosing, treatment duration, and patient selection criteria must be precisely defined. Second, the heterogeneity of PAI-1 expression among different tumor types and within distinct TME subtypes may necessitate biomarker-driven stratification to identify patients most likely to benefit. Third, resistance mechanisms to PAI-1 blockade, including compensatory pathways in stromal cells, require further elucidation.

Future research should focus on integrating PAI-1 inhibition into rational combination regimens, such as pairing with ICIs, anti-angiogenic agents, or stroma-targeting therapies, to enhance therapeutic synergy. Longitudinal studies incorporating spatial transcriptomics and single-cell profiling will be critical to map dynamic changes in the TME during PAI-1 blockade and to refine predictive biomarkers. In addition, the exploration of PAI-1’s role in the interplay between SASP factors and ECM remodeling may reveal novel intervention points.

Ultimately, advancing PAI-1-targeted strategies from experimental therapy to routine clinical practice will depend on well-designed, adequately powered trials that account for the biological diversity of skin malignancies. If successful, such approaches may offer a new avenue to overcome immune resistance, reduce metastatic spread, and improve survival outcomes for patients with otherwise refractory disease.

## 5. Conclusions

PAI-1 has emerged as a pivotal stromal mediator in the pathogenesis and progression of skin malignancies. Beyond its canonical role in fibrinolysis, PAI-1 orchestrates a wide array of tumor-supportive mechanisms, including immunosuppression, angiogenesis, extracellular matrix remodeling, and resistance to immune checkpoint inhibitors and cytotoxic chemotherapy. Through its regulation of TAMs, CAFs, and endothelial cells, PAI-1 sustains an immunosuppressive tumor microenvironment and promotes cancer progression across melanoma, cSCC, CAS, and MF (CTCL).

Recent preclinical studies and early-phase clinical trials—particularly those involving the small-molecule inhibitor TM5614—demonstrate that targeting PAI-1 can enhance anti-PD-1 antibody efficacy, suppress stromal-driven resistance mechanisms, and induce durable antitumor effects. However, the pleiotropic and context-dependent functions of PAI-1 necessitate careful evaluation of dosing strategies, patient stratification, and potential compensatory mechanisms within the tumor stroma. Furthermore, the heterogeneity of PAI-1 expression across tumor subtypes underscores the importance of biomarker-driven approaches to identify patients most likely to benefit from PAI-1 inhibition. Looking ahead, rational combination regimens incorporating PAI-1 inhibitors with immune checkpoint blockade, anti-angiogenic therapies, or stroma-targeted interventions may hold the greatest promise. Integration of high-resolution technologies such as spatial transcriptomics and single-cell profiling will be critical to unravel the dynamic interplay between PAI-1, SASP factors, and extracellular matrix remodeling. Collectively, advancing PAI-1-directed therapies from experimental strategies to clinical application represents a compelling opportunity to overcome immune resistance, mitigate metastatic progression, and improve survival outcomes for patients with otherwise refractory skin cancers.

## 6. Limitations

The clinical trials aiming to develop novel therapies targeting PAI-1 have so far been based solely on exploratory or preclinical data, with inherent limitations in sample size and follow-up duration. Moreover, since all cutaneous malignancies under investigation are rare cancers, stratification remains highly challenging due to difficulties in securing a sufficient number of cases.

## Figures and Tables

**Table 1:** Summary for tumor-promoting effects of PAI-1.

	Tumor Promoting Effects of PAI-1		Possible Add-On Medication for TM5614
Melanoma	increase	PD-L1	nivolumab
		sPD-L1	
		ratio of Tregs	pembrolizumab
	decrease	number of TAMs	
		number of CAFs	other anti-PD-1 Ab
		cytotoxic function of T cells	
cSCC	induced by	p53 mutant	cisplatin
		Smad 2/3 signal	
	activate	p53/p21 pathway	oxaliplatin
		LRP1/p65 signal	
	increase	IL-6, IL-8, other SASP	radioimmunotherapy
		MMPs	
CAS	increase	IL-23p19, VEGF-C, CXCL5	paclitaxel
		VEGF signal	
	activate	Fas-L mediated apoptosis	pazopanib
		p53/p21 signal	
MF	induced by	hyalronic acid mediated SASP	bexarotene

## Data Availability

No new data were created or analyzed in this study. Data sharing is not applicable to this article.

## Abbreviations

The following abbreviations are used in this manuscript:

AD	Atopic dermatitis
CAFs	Cancer associated fibroblasts
CAS	Cutaneous angiosarcoma
cSCC	Cutaneous squamous cell carcinoma
ECs	Endothelial cells
ECM	Extracellular matrix
HA	Hyaluronic acid
HS	Hidradenitis suppurativa
ICIs	Immune checkpoints inhibitors
MMP	Matrix metalloproteinase
MF	Mycosis fungoides
PAI-1	plasminogen activator inhibitor-1
PD-L1	programmed cell death ligand 1
SASP	Senescence-associated secretory phenotype
TAMs	tumor-associated macrophage
TKI	Tyrosine kinase inhibitor
TME	Tumor microenvironment
